# Serial ultrasound assessment of flexor carpi ulnaris and biceps brachii cross-sectional area in critically ill patients: A prospective cohort study

**DOI:** 10.1371/journal.pone.0361123

**Published:** 2026-10-29

**Authors:** Keiichiro Aoki, Fumihito Kasai, Kazuki Komaba, Jin Saito, Akira Yoshikawa, Naonori Tashiro, Hiroyasu Inoue, Kengo Uchibori, Miwa Fukazawa

**Affiliations:** 1 Department of Occupational Therapy, School of Rehabilitation, Tokyo Professional University of Health Sciences, Koto-ku, Tokyo, Japan; 2 Division of Occupational Therapy, Department of Rehabilitation, School of Nursing and Rehabilitation Sciences, Showa Medical University, Midori-ku, Kanagawa, Japan; 3 Department of Rehabilitation Medicine, Showa Medical University, School of Medicine, Shinagawa-ku, Tokyo, Japan; 4 Division of Morphology, Anatomy and Physiology, Department of Medical Basics, Specialty and Education, Showa Medical University, Graduate School of Nursing and Rehabilitation Sciences, Midori-ku, Kanagawa, Japan; 5 Department of Physical Therapy, Faculty of Rehabilitation, Gunma Paz University, Takasaki-shi, Japan; 6 Division of Cardiopulmonary Rehabilitation Science, Department of Rehabilitation, Showa Medical University, Graduate School of Nursing and Rehabilitation Sciences, Midori-ku, Kanagawa, Japan; Università degli Studi di Milano: Universita degli Studi di Milano, ITALY

## Abstract

**Background:**

Serial ultrasound measurements may help characterize muscle wasting in critically ill patients. The flexor carpi ulnaris (FCU) is a superficial forearm muscle that may provide an additional assessment site. We described longitudinal FCU and biceps brachii (BB) cross-sectional area (CSA) changes and explored between-muscle differences and clinical associations.

**Methods:**

This prospective two-center observational study enrolled 23 intensive care unit (ICU) patients; 20 were included in the analysis (median age, 57 years; range, 33–82). CSA was measured on days 0, 5, 7, and 14 after ICU admission. Analyses used Friedman tests, exact conditional Wilcoxon signed-rank tests with Bonferroni correction, and exploratory Spearman correlations. Post hoc between-muscle comparisons assessed participant-level percentage changes.

**Results:**

Mean FCU CSA decreased from 1.36 to 1.08 cm² and mean BB CSA from 6.08 to 5.09 cm² over 14 days, corresponding to reductions of 20.9% and 16.4% in the unrounded group means. The median paired difference in percentage change (FCU minus BB) was −11.9 percentage points on day 7 (unadjusted p = 0.0023), meeting the Bonferroni threshold of 0.05/3. Differences on days 5 and 14 did not meet this threshold (p = 0.1429 and p = 0.2024). Baseline CSA values were correlated (ρ = 0.845, p < 0.001), as were 14-day percentage changes (ρ = 0.549, p = 0.0122); correlation p-values were unadjusted.

**Conclusions:**

Serial ultrasound documented decreases in FCU and BB CSA in this small cohort, with an exploratory direct comparison identifying a greater proportional FCU decrease on day 7. These findings support further evaluation of FCU ultrasound as an additional muscle-monitoring measure. Its value for earlier detection of muscle wasting or diagnosis of ICU-acquired weakness requires direct validation.

## Introduction

In critically ill patients admitted to the intensive care unit (ICU), rapid progression of skeletal muscle loss and muscle weakness is known to occur from the acute phase—these conditions are recognized as key components of post-intensive care syndrome [1]. In particular, ICU-acquired weakness (ICU-AW)—defined as muscle weakness newly developed during the ICU stay—has been reported to have long-term adverse effects on physical function and health-related quality of life after discharge from the ICU [2,3].

ICU-AW is commonly assessed using the Medical Research Council (MRC) score, administered by healthcare professionals such as physical and occupational therapists involved in early rehabilitation [4]. However, the assessment requires patient wakefulness and cooperation, which often makes it difficult to perform in the early ICU phase [5]. Therefore, ultrasound-based measurement of muscle cross-sectional area (CSA) has attracted attention as a more objective and non-invasive method for assessing muscle mass [6].

Previous studies have documented ultrasound-detected muscle loss during critical illness, including changes in the rectus femoris and BB [7,8]. Measurement protocols, anatomical sites, patient populations, and observation periods differ across studies, limiting direct comparison of the magnitude of change [6,9]. At the bedside, patient positioning and the location of dressings, vascular access, and life-support equipment may also affect access to a particular muscle. These practical considerations motivate evaluation of additional measurement sites alongside established approaches and broader rehabilitation assessment [10].

The FCU lies superficially in the medial forearm and can be imaged with the elbow flexed and the forearm in a neutral position. Its anatomical location provides a rationale for investigating it as an additional ultrasound assessment site. Characterizing FCU CSA alongside BB CSA may help determine whether measurements at these two sites capture similar patterns of muscle change during critical illness.

This prospective cohort study aimed to characterize FCU and BB CSA trajectories during the first 14 days after ICU admission and to explore associations between their baseline sizes, longitudinal changes, and selected clinical variables.

## Materials and methods

### Study design and ethics

This prospective observational study was conducted at Showa Medical University Hospital and Showa Medical University Fujigaoka Hospital. The study was conducted in accordance with the principles of the Declaration of Helsinki, and the protocol was approved by the Institutional Review Board of Showa Medical University (Approval No. 2023–041-B). Participant recruitment began on 22/03/2024 and ended on 06/02/2025. This was an observational study without random allocation to interventions or muscle-assessment groups.

As patients in the ICU are often in a critical condition upon admission, obtaining written informed consent in advance was frequently impractical. Therefore, considering that the ultrasound measurements were part of routine clinical care, verbal informed consent for study participation was obtained from the patient or their legally authorized representative (e.g., family member) once the patient’s condition stabilized or communication became feasible. The content of the consent was documented in the medical records. Since ultrasound measurements were performed as part of standard care, consent was occasionally obtained 1–2 days after the initial measurements. These consent procedures, including verbal consent and its timing, were approved by the Institutional Review Board.

### Participants

The inclusion criteria were as follows: (1) patients registered within 24 hours of ICU admission, (2) patients judged by the attending physician to require ≥48 hours of mechanical ventilation, (3) patients with a predicted ICU stay of ≥5 days, and (4) patients aged ≥18 years. Patients were excluded from the study according to the following exclusion criteria: (1) presence of limb trauma or amputation, (2) diagnosis of primary neuromuscular disease, or (3) inability to acquire clear ultrasound images for measurement.

### Sample size

The study had a recruitment target of 25 participants

### Ultrasound measurement protocol

CSA was measured using a SONIMAGE MX1 ultrasound system (Konica Minolta, Tokyo, Japan) with a linear probe (L11-3). Ample contact gel was used to avoid compression of the muscle by the probe, which was consistently placed perpendicular to the longitudinal axis of the limb.

The measurement sites and postures were as follows: the FCU was measured at the midpoint between the olecranon and ulnar styloid, with the patient lying in a supine position, the elbow flexed at 90°, and the forearm in a neutral position; and the BB was measured at the point two-thirds distal along the line from the acromion to the cubital fossa crease, with the patient lying in a supine position with the elbow extended.

After clear ultrasound images were obtained, the CSA was calculated by tracing the outer boundary of the muscle belly (Fig 1a, 1b). To ensure reproducibility, measurement sites were marked on the skin, and all measurements were taken from the same site. Each muscle was measured twice consecutively by a single evaluator, and the mean value was used for analysis.

**Fig 1:**
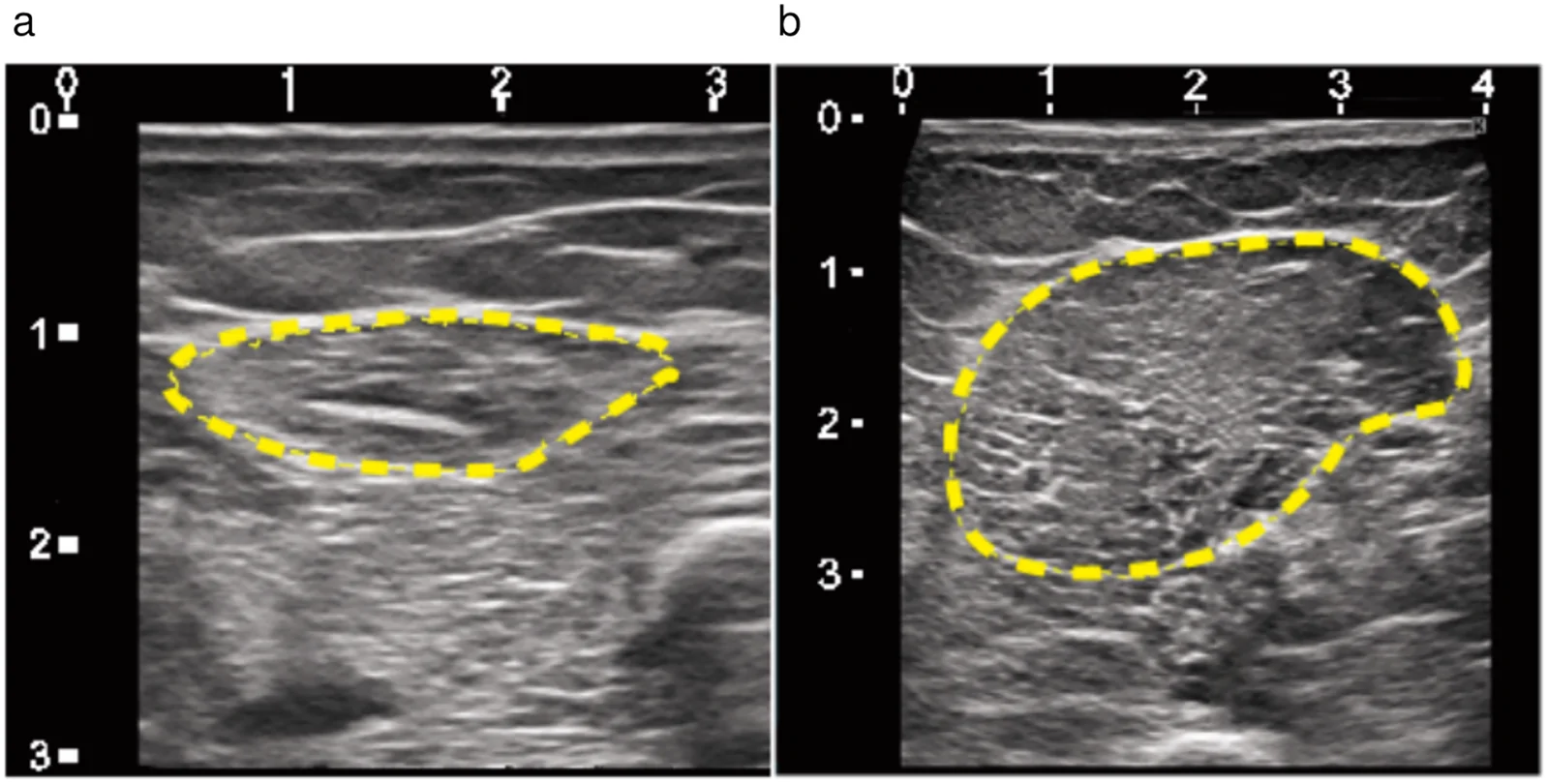
Representative ultrasound images of the FCU and BB. (a) FCU at the midpoint between the olecranon and ulnar styloid, with the elbow flexed at 90° and the forearm neutral. (b) BB at two-thirds of the distance from the acromion to the cubital fossa crease, with the elbow extended. The yellow dashed outlines indicate the traced muscle boundaries used to obtain CSA. Abbreviations: FCU, flexor carpi ulnaris; BB, biceps brachii; CSA, cross-sectional area.

### Reliability of ultrasound measurements

Before the main study, two examiners assessed measurement reliability in five healthy adults (three men and two women; age range, 24–36 years). Each examiner measured each muscle twice. Inter- and intra-rater intraclass correlation coefficients (ICCs) were used to summarize reliability in this separate sample (Tables 1 and 2). These estimates were not obtained in critically ill patients.

**Table 1:** Summary statistics for inter-rater reliability testing of CSA measurement using ultrasound.

	Flexor Carpi Ulnaris		Biceps Brachii	
ID	Mean (cm²)	SD	Mean (cm²)	SD
No. 1	1.59	0.01	8.00	0.76
No. 2	0.91	0.01	4.45	0.22
No. 3	1.14	0.03	5.09	0.66
No. 4	1.46	0.04	7.65	0.75
No. 5	1.14	0.05	6.00	0.66

Abbreviations: SD, standard deviation

**Table 2:** ICCs for the ultrasound measurement of the FCU and BB CSAs.

Muscle	Comparison	ICC	Interpretation
FCU	Inter-rater	0.98	Excellent
FCU	Intra-rater (Examiner A)	0.99	Excellent
FCU	Intra-rater (Examiner B)	0.99	Excellent
BB	Inter-rater	0.96	Excellent
BB	Intra-rater (Examiner A)	0.97	Excellent
BB	Intra-rater (Examiner B)	0.98	Excellent

Abbreviations: FCU, flexor carpi ulnaris; BB, biceps brachii; ICC, intraclass correlation coefficient

### Evaluation items and outcomes

The primary outcome of this study was the longitudinal change in the CSA of the FCU and BB during the ICU stay. Measurements were performed at four time points: day 0 (within 24 hours of ICU admission), day 5, day 7, and day 14. These time points were set with reference to the assessment schedule used by Zhang et al. [11], adjusted for feasibility and clinical workflow at the two participating institutions.

Clinical and rehabilitation characteristics were extracted from medical records, including ICU and hospital length of stay, Sequential Organ Failure Assessment (SOFA) score, duration of mechanical ventilation, Richmond Agitation-Sedation Scale, time to rehabilitation initiation, Functional Status Score for the ICU (FSS-ICU), Barthel Index (BI), and discharge outcome. FSS-ICU and BI were collected within one week of rehabilitation initiation. These measures describe the cohort; only the associations specified below were analyzed inferentially.

### Statistical analysis

Statistical analyses used JMP Pro (version 16, SAS Institute Inc., Cary, NC, USA) and Python with SciPy 1.17.0. The reported serial CSA tests and Spearman correlations were calculated using Python. Statistical analysis was performed without blinding. The public analysis data and descriptive clinical characteristics, excluding participant identifiers and individual ages, are provided in S1 Dataset. All tests were two-sided where applicable. Bonferroni correction was applied separately to the six temporal comparisons within each muscle (threshold 0.05/6 = 0.00833) and to the three exploratory between-muscle comparisons (threshold 0.05/3 = 0.01667). Reported p-values are unadjusted unless explicitly labeled otherwise.

### Primary analysis

Within-muscle changes across days 0, 5, 7, and 14 were assessed using Friedman tests with tie correction and chi-square approximation. Follow-up Wilcoxon signed-rank comparisons used exact conditional sign-randomization distributions of the nonzero differences, with midranks for tied absolute differences; zero differences were omitted. Two-sided p-values were twice the smaller inclusive tail probability, capped at 1. Differences were rounded to 10 decimal places before ranking to remove floating-point artifacts. The same signed-rank procedure was used for all within- and between-muscle comparisons.

Post hoc exploratory comparisons assessed differences in participant-level percentage changes between FCU and BB. For each participant, percentage change was 100 × (follow-up CSA − baseline CSA)/baseline CSA. At each follow-up, the paired difference was FCU percentage change minus BB percentage change; negative values indicate a larger proportional FCU decrease. Sample medians summarize these differences. Signed-rank inference assumes symmetry of the paired differences under the null. These analyses compare cumulative changes at sampled time points. Table 4 separately describes changes in unrounded group means. No values were imputed.

### Exploratory correlation analyses

Spearman correlations assessed FCU versus BB CSA at baseline and their participant-level percentage changes from day 0 to day 14. Exploratory correlations also assessed BMI and SOFA score with baseline FCU CSA; baseline BB CSA with BB percentage change; and age, SOFA score, duration of mechanical ventilation, and baseline FCU CSA with FCU percentage change. These nine correlations are hypothesis-generating; their two-sided asymptotic p-values are reported without multiplicity correction. BMI sensitivity was assessed by repeating the same correlation after omitting the participant with BMI 53.13 kg/m².

## Results

### Reliability of ultrasound measurements

In the five healthy volunteers, the inter-rater ICC was 0.98 for FCU and 0.96 for BB. Intra-rater ICCs were 0.99 for FCU for both examiners and 0.97 and 0.98 for BB for Examiners A and B, respectively (Tables 1 and 2).

### Participant characteristics

Twenty-three participants were enrolled against a target of 25. Three were excluded because ultrasound images could not be acquired in accordance with the study protocol, leaving 20 participants (10 men and 10 women) in the analysis. The excluded participants were aged 29–73 years (two men and one woman), with SOFA scores of 5–12 and mechanical ventilation durations of 5–13 days. Retained characteristics and exclusion reasons are provided in S1 Dataset. The analyzed participants had a median age of 57 years (range, 33–82), a median SOFA score of 8.5 (range, 0–15), and a median mechanical ventilation duration of 12.5 days (range, 5–70) (Table 3). All eight serial CSA measurements were available for each analyzed participant, yielding 160 values in total.

**Table 3:** Participant characteristics.

Characteristics	(N = 20)
Age, years	57 (33–82)
Sex	
Male	10 (50)
Female	10 (50)
Primary Diagnosis	
Infectious/Septic Conditions	7 (35)
Neurological Disorders/Post-Cardiopulmonary Arrest	5 (25)
Cardiovascular Diseases	4 (20)
Respiratory/Metabolic Disorders	2 (10)
Other	2 (10)
Height, cm	167 (145–190)
Weight, kg	62.65 (44.5–169.1)
BMI, kg/m²	22.68 (16.46–53.13)
ICU length of stay, days	18 (9–74)
Hospital length of stay, days	45.5 (16–115)
SOFA score	8.5 (0–15)
Duration of mechanical ventilation, days	12.5 (5–70)
RASS score	−5 (−5–1)
Time to rehabilitation initiation, days	1.5 (1–14)
FSS-ICU	1 (1–35)
BI	0 (0–75)
Discharge outcome	
Discharged home	7 (35)
Transferred	8 (40)
Deceased	5 (25)

Data are presented as the median (range) or n (%)

Abbreviations: BMI, body mass index; ICU, intensive care unit; SOFA, Sequential Organ Failure Assessment; RASS, Richmond Agitation-Sedation Scale; FSS-ICU, Functional Status Score for the Intensive Care Unit; BI, Barthel Index

### Temporal changes in muscle CSA

Mean FCU CSA decreased from 1.36 ± 0.46 cm² at baseline to 1.19 ± 0.42, 1.11 ± 0.39, and 1.08 ± 0.40 cm² on days 5, 7, and 14, respectively. Mean BB CSA was 6.08 ± 2.54, 5.69 ± 2.73, 5.71 ± 2.60, and 5.09 ± 2.40 cm² at the corresponding time points (Table 4). Percentage changes were calculated before rounding these means; the day-14 reductions were 20.9% for FCU and 16.4% for BB.

**Table 4:** Temporal changes in muscle cross-sectional area.

Measure	Day 0	Day 5	Day 7	Day 14
FCU CSA, cm²	1.36 ± 0.46	1.19 ± 0.42	1.11 ± 0.39	1.08 ± 0.40
BB CSA, cm²	6.08 ± 2.54	5.69 ± 2.73	5.71 ± 2.60	5.09 ± 2.40
FCU change, %	Reference	−12.68	−18.31	−20.92
BB change, %	Reference	−6.40	−6.13	−16.37

CSA values are mean ± SD in cm² (n = 20 at every time point). Percentage changes are descriptive changes in the unrounded group means, calculated as 100 × (follow-up mean − baseline mean)/baseline mean; calculating them from the rounded displayed means will not reproduce them exactly. These are distinct from participant-level percentage changes used in the paired tests and correlations. Abbreviations: FCU, flexor carpi ulnaris; BB, biceps brachii; CSA, cross-sectional area; SD, standard deviation.

Friedman tests indicated changes over time in both muscles (FCU: χ²(3) = 37.50, p < 0.0001; BB: χ²(3) = 22.38, p < 0.0001). At the Bonferroni threshold of 0.00833, FCU reductions from baseline were significant on days 5, 7, and 14 (all unadjusted p < 0.0001), as was the day-5 versus day-14 comparison (p = 0.0062). FCU day-5 versus day-7 (p = 0.0136) and day-7 versus day-14 (p = 0.2124) comparisons did not meet the corrected threshold. For BB, baseline versus day-14 (p = 0.0012), day-5 versus day-14 (p = 0.0035), and day-7 versus day-14 (p = 0.0042) comparisons met the threshold; baseline versus day-5 (p = 0.0742), baseline versus day-7 (p = 0.0637), and day-5 versus day-7 (p = 0.3730) comparisons did not (Fig 2).

**Fig 2:**
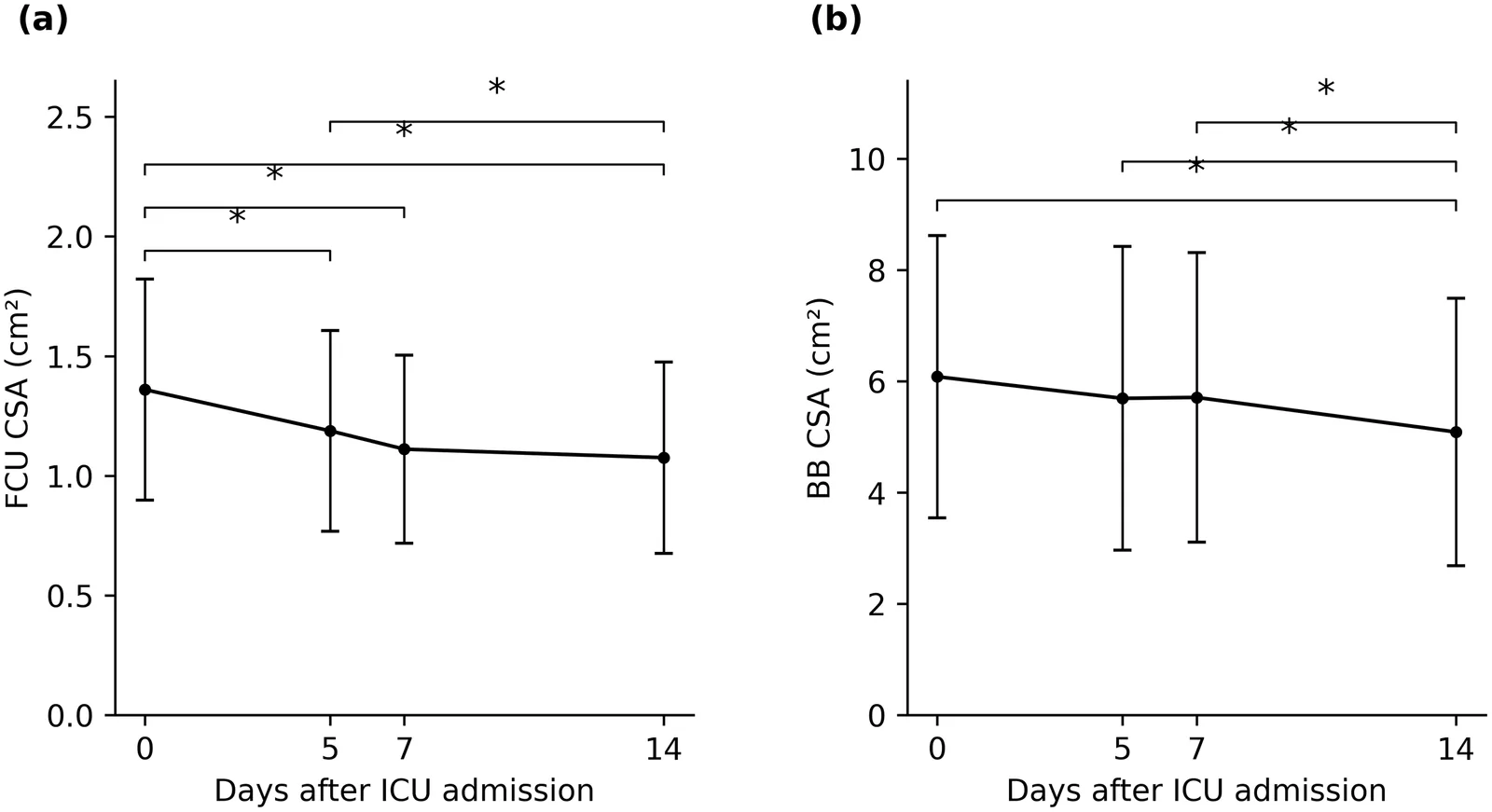
Serial FCU and BB CSA measurements. (a) FCU and (b) BB mean CSA with SD error bars on days 0, 5, 7, and 14 after ICU admission (n = 20 at each time point). Brackets with asterisks identify within-muscle comparisons meeting the Bonferroni threshold of 0.00833 in two-sided exact conditional Wilcoxon signed-rank tests. FCU comparisons meeting this threshold are day 0 versus days 5, 7, and 14, and day 5 versus day 14; BB comparisons are days 0, 5, and 7 versus day 14. Both axes start at zero, with separate vertical scales for the two muscles. Direct between-muscle tests use participant-level percentage changes and are reported in the Results, not by these brackets.

In the direct exploratory comparisons, median paired differences in participant-level percentage change (FCU minus BB) were −5.6 percentage points on day 5 (p = 0.1429), −11.9 on day 7 (p = 0.0023), and −8.0 on day 14 (p = 0.2024). Only day 7 met the Bonferroni threshold of 0.01667 (adjusted p = 0.0070).

### Correlation between the CSAs of the FCU and BB

Baseline FCU and BB CSA values were positively correlated (Spearman’s ρ = 0.8447, p < 0.0001; Fig 3a). Their participant-level percentage changes from day 0 to day 14 were also positively correlated (ρ = 0.5489, p = 0.0122; Fig 3b). These unadjusted correlation results are exploratory.

**Fig 3:**
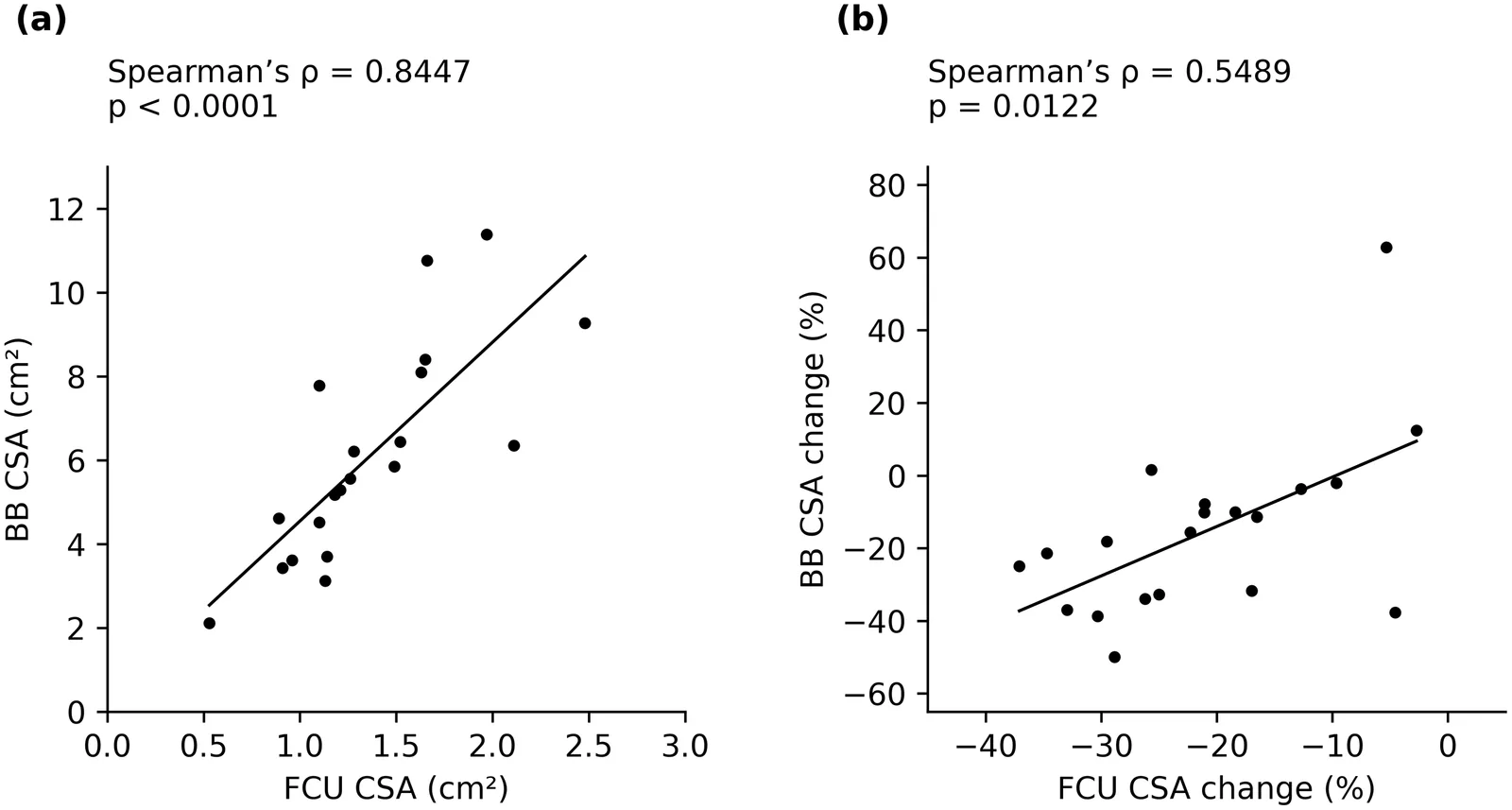
Exploratory correlations between FCU and BB CSA measures (n = 20). (a) Baseline absolute CSA values (Spearman’s ρ = 0.8447, p < 0.0001). (b) Participant-level percentage changes from day 0 to day 14 (ρ = 0.5489, p = 0.0122). Plotted p-values are two-sided, asymptotic, and unadjusted. The straight lines are ordinary least-squares fits displayed as descriptive visual guides; they are not fitted Spearman models. These associations do not establish agreement or interchangeability between muscles. Abbreviations: FCU, flexor carpi ulnaris; BB, biceps brachii; CSA, cross-sectional area.

### Exploratory associations with baseline measurements and clinical variables

Exploratory correlations were as follows: BMI with baseline FCU CSA (ρ = 0.335, p = 0.149); SOFA score with baseline FCU CSA (ρ = −0.121, p = 0.612); baseline BB CSA with BB percentage change (ρ = −0.011, p = 0.965); age with FCU percentage change (ρ = 0.034, p = 0.887); SOFA score with FCU percentage change (ρ = 0.158, p = 0.505); mechanical ventilation duration with FCU percentage change (ρ = 0.028, p = 0.907); and baseline FCU CSA with FCU percentage change (ρ = −0.007, p = 0.977). None had an unadjusted p-value below 0.05. Excluding the participant with BMI 53.13 kg/m² reduced the BMI–baseline FCU correlation from ρ = 0.335 (n = 20, p = 0.149) to ρ = 0.224 (n = 19, p = 0.357).

## Discussion

In this prospective two-center cohort, serial ultrasound measurements documented reductions in both FCU and BB CSA over 14 days. Mean FCU CSA decreased by 20.9% and mean BB CSA by 16.4%. The direct exploratory comparison identified a greater proportional FCU decrease on day 7 after correction for three comparisons. By describing FCU alongside BB, this study extends the characterization of upper-limb muscle changes during critical illness and provides a basis for evaluating the FCU as an additional ultrasound monitoring site.

The day-7 comparison suggests that the magnitude of proportional CSA change can differ between the two muscles at a sampled time point. Differences on days 5 and 14 did not meet the corrected threshold, which should not be interpreted as equivalence. The distinction between within-muscle and between-muscle tests is essential: a significant FCU change and a nonsignificant BB change on day 5 do not themselves demonstrate a difference between muscles. Because measurements were obtained only on days 0, 5, 7, and 14, the nine-day separation between the first significant within-muscle tests reflects the sampling and testing schedule rather than an estimated biological lead time. The data characterize cumulative changes at the observed time points without identifying atrophy onset or a muscle-by-time interaction.

Positive correlations between baseline CSAs and between 14-day percentage changes indicate related patterns of muscle size and change within this cohort. Together with the day-7 comparison, these findings suggest that measurements at the two sites may capture both shared and muscle-specific features of the response to critical illness. The decreases in CSA are consistent in direction with previous ICU ultrasound studies [6–9], although differences in protocols and populations limit quantitative comparisons. Correlation alone does not establish agreement or interchangeability, and the change correlation remains exploratory because its p-value was not adjusted for multiple correlation tests.

Several biological processes could contribute to the observed CSA trajectories, including inflammation, altered metabolism, immobilization, treatment exposures, and fluid shifts [12,13]. Fiber-type composition may contribute to muscle-specific responses. McIntosh et al. examined fiber-type composition in monkey forearm muscles [14]; however, extrapolation to differences between human FCU and BB remains uncertain. As fiber composition and these other mechanisms were not measured, their contributions remain hypotheses for future investigation.

The FCU’s superficial forearm location and the serial measurements obtained in the analyzed cohort provide a practical rationale for further bedside evaluation. The high ICC point estimates in five healthy volunteers also provide preliminary evidence of reproducibility under those test conditions. Translation to routine ICU monitoring requires assessment under the positioning constraints, edema, and image-quality limitations encountered in critically ill patients. In particular, comparative acquisition time, interference from devices, and image-acquisition success should be measured directly. Linking CSA changes to strength and functional outcomes would help define the clinical contribution of this local measure of muscle size.

The exploratory analyses did not identify clear associations of FCU measurements with BMI, clinical severity, ventilation duration, or baseline muscle size. These estimates are imprecise in a cohort of 20 and should guide the design of larger studies rather than be interpreted as evidence that the clinical factors are unimportant. Future analyses should address confounding and the timing of clinical measurements.

Several limitations should be considered. First, the recruitment target of 25 was not reached, and the final sample of 20 limits precision and statistical power, particularly for exploratory comparisons and correlations. Second, three participants were excluded for protocol-related image-acquisition difficulties; their small number prevents a reliable assessment of selection bias. Third, site-level differences in case mix and care were not accounted for. Fourth, the direct between-muscle comparisons were post hoc, and multiplicity was controlled within specified test families rather than across the study; the correlation p-values were unadjusted. Fifth, sparse sampling limits temporal interpretation, and small CSA fluctuations, such as the BB increase between days 5 and 7, may reflect fluid shifts or measurement variability as well as biological change. Sixth, reliability was evaluated in healthy volunteers rather than ICU patients, and no direct validation against MRC sum scores or handgrip strength was performed. The study therefore does not establish diagnostic accuracy for ICU-acquired weakness or an individual-patient change threshold. Finally, the absence of measurements of fiber-type composition, inflammatory biomarkers, nutritional parameters, and corticosteroid exposure limits mechanistic interpretation.

The principal contribution is the longitudinal description of FCU CSA alongside BB CSA in critically ill patients, including an exploratory difference in proportional change on day 7. This provides a foundation for studies that combine reliable ICU bedside measurements, denser follow-up, and strength and functional assessments to determine the value of adding FCU ultrasound to muscle monitoring.

## Conclusions

FCU and BB CSA decreased over 14 days in this prospective cohort. An exploratory direct comparison identified a greater proportional FCU decrease on day 7, whereas differences on days 5 and 14 did not meet the corrected threshold. These findings support further evaluation of FCU ultrasound as an additional site for monitoring muscle changes in critically ill patients. Its ability to improve early detection or clinical assessment of ICU-acquired weakness requires direct validation.

## Supporting information

S1 Dataset De-identified serial FCU and BB CSA measurements and clinical covariates for the 20 analyzed participants (Patients20 and Clinical20), characteristics of the three excluded participants (Excluded3), and the individual healthy-volunteer measurement summaries from Table 1 (Healthy5_Summary). Participant identifiers and individual ages are omitted. Readme and Dictionary describe units, coding and provenance. The workbook does not include examiner-level repeated reliability measurements or individual diagnosis-category assignments.
(XLSX)

S1 Checklist STROBE statement checklist. (DOCX)
